# Retropharyngeal Abscess in an Adult Patient Presenting with Neck Fullness and Dysphagia: A Case Report

**DOI:** 10.21980/J8M36G

**Published:** 2025-01-31

**Authors:** Justin Rederer, Tanner Folster, Sara Dimeo

**Affiliations:** * Midwestern University Arizona College of Osteopathic Medicine, Glendale, AZ; ^ Dignity Health Chandler Regional Medical Center, Department of Emergency Medicine, Chandler, AZ

## Abstract

Retropharyngeal abscess (RPA) is an uncommon yet potentially life-threatening condition that is more often seen in young children and may be misdiagnosed in adults presenting with atypical features.^1^ Retropharyngeal abscess results from spread of antecedent upper respiratory tract infection or traumatic inoculation via foreign body ingestion or medical instrumentation. Clinically, RPA may present with fever, pharyngitis, neck pain, and dysphagia. Diagnosis is often confirmed with imaging studies. We present a case of a 66-year-old female with asthma, hypertension, and gastroesophageal reflux disease (GERD) who presented to the emergency department (ED) for evaluation of neck fullness, shoulder pain, dysphagia, and abdominal pain starting less than 24 hours prior to presentation. Computed tomography (CT) revealed a prevertebral/retropharyngeal fluid collection from the odontoid tip to the C4 vertebral body measuring 5.4 × 1.0 × 3.3 centimeters (cm) in size with associated edema at the left neck base extending into the upper chest, suggestive of retropharyngeal abscess. The patient received intravenous (IV) vancomycin and piperacillin/tazobactam and was transferred to a higher level of care for otolaryngologist evaluation. The patient remained stable without airway compromise while in our department. This case underscores challenges in diagnosing atypical presentations of RPA in adults, emphasizing timely recognition to prevent complications.

**Topics:**

Dysphagia, retropharyngeal abscess, prevertebral abscess, otolaryngology.

**Figure:**
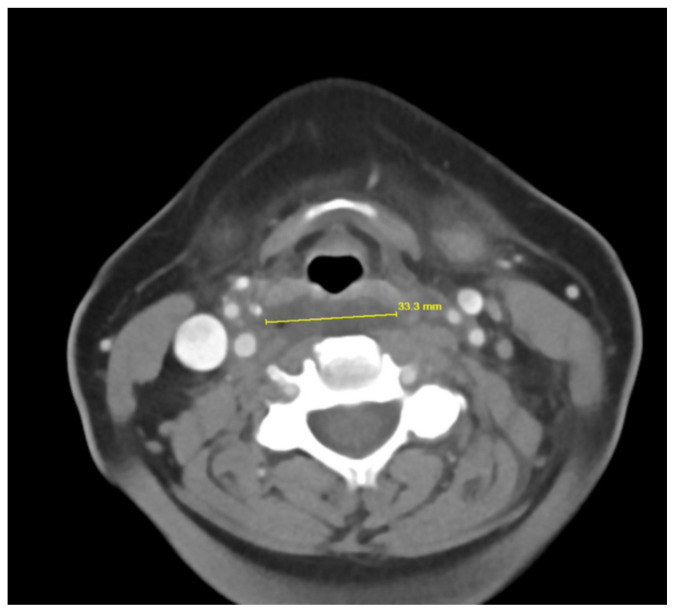


**Figure:**
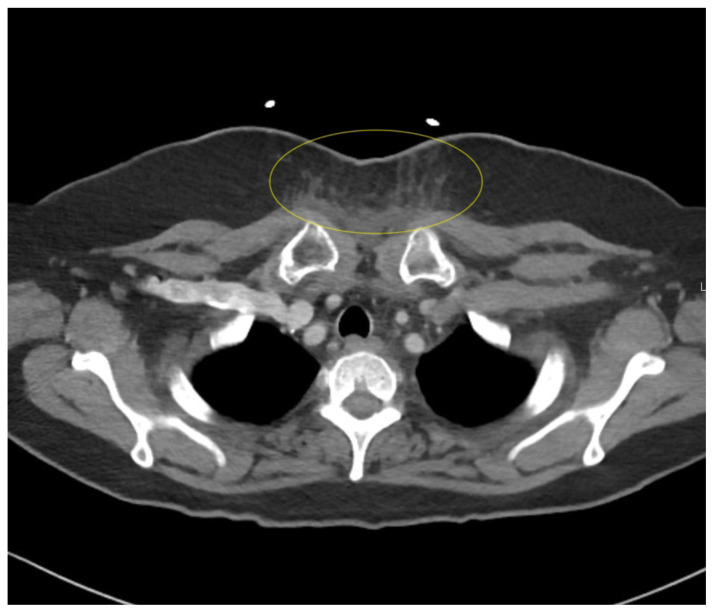


**Figure:**
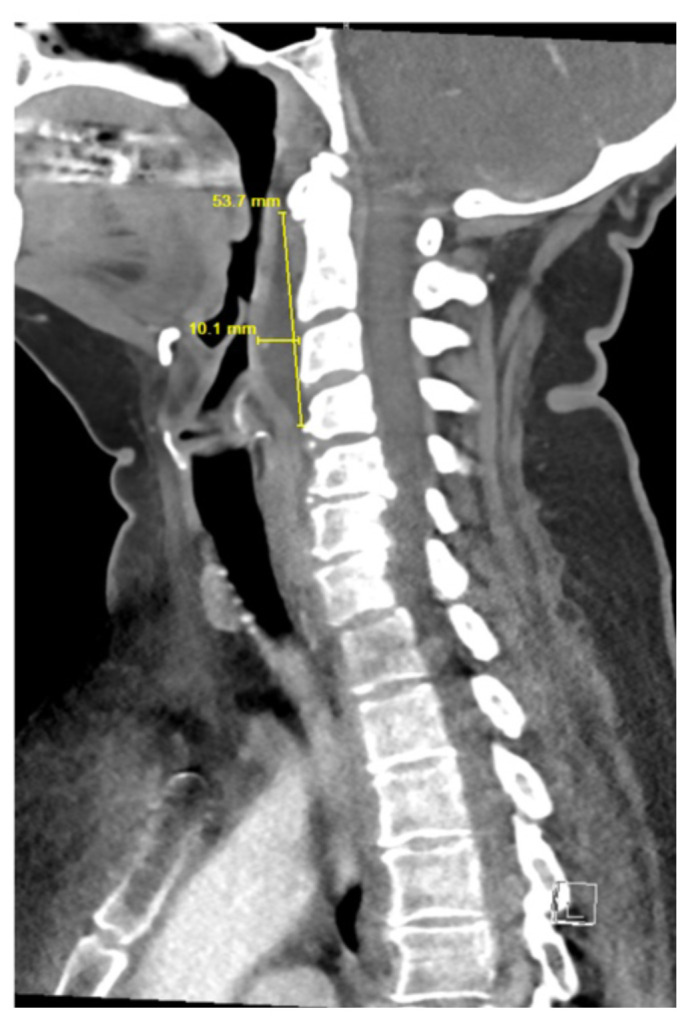


## Brief Introduction

Retropharyngeal abscess is a relatively uncommon but potentially life-threatening suppurative infection of the deep neck space, which is a potential space that exists between the posterior pharynx and prevertebral fascia, and spans the length between the skull base and the posterior mediastinum. Though RPA tends to occur more frequently in children under five years of age, it can also be observed in adults.^1^ In children, it is believed that contiguous spread of an antecedent upper respiratory tract or oropharyngeal infection may seed the lymphatics present within the retropharyngeal space, leading to abscess, cellulitis, and/or phlegmon formation. Since these lymphatics tend to involute by early adolescence, RPA is not thought to occur in adults via this same mechanism.^2^ Instead, it is believed RPA in older children and adults is more likely to stem from local trauma to the posterior pharyngeal wall from ingested foreign body or via medical instrumentation. Complications of RPA may include airway obstruction, acute necrotizing mediastinitis, necrotizing fasciitis, erosion into the carotid artery, Lemierre’s Syndrome (jugular thrombophlebitis), epidural abscess, pericarditis, aspiration pneumonia, and bacteremia/sepsis.^2^ Clinically, the presentation of RPA is variable; however, most patients tend to be ill-appearing with fever and possible pharyngitis, neck pain, neck swelling, dysphagia, odynophagia, drooling, trismus, voice changes, and/or respiratory distress. Extension of the infection into the posterior mediastinum may present with chest pain. Physical exam may be remarkable for neck asymmetry, bulging of the posterior oropharynx, torticollis, limited neck extension, and/or cervical lymphadenopathy; however, early presentations may be difficult to differentiate from uncomplicated pharyngitis.

## Presenting concerns and clinical findings

A 67-year-old female with a past medical history of gastroesophageal reflux disease (GERD), hypertension, and asthma presented to the ED by private vehicle with a chief complaint of left-sided shoulder and neck “fullness” as well as some pain with swallowing. She noted some slight subjective wheezing associated with this. Her symptoms were non-exertional, and she had no new foods, exposures, or medications. She had taken oral diphenhydramine the evening before with no improvement, prompting her visit. On arrival, the patient’s vitals included a temperature of 37.1°C, a heart rate of 89, blood pressure of 189/87, respiratory rate of 18, and oxygen saturation of 98% on room air. On examination, the patient was a pleasant female who was lying supine on an exam gurney in no acute distress, though with a slightly anxious affect. A full physical examination yielded normal head, ears, eyes, nose and throat, abdominal, cardiac, pulmonary, and skin examinations, except for very faint wheezing in bilateral lung fields and a faintly erythematous appearance to the skin over her chest and anterior neck. Specifically, she had no cervical lymphadenopathy, and her uvula was midline with no pharyngeal erythema or exudate. She was phonating normally without any sign of stridor or dysphonia. Lab work was drawn, including complete blood count, comprehensive metabolic panel, lipase and troponin. An electrocardiogram (ECG) was also obtained. Computed tomography (CT) imaging of the neck and chest with contrast were also ordered. For symptomatic relief of an initially suspected allergic reaction versus pharyngitis, the patient was given dexamethasone, diphenhydramine, and a single dose of nebulized albuterol-ipratropium.

## Significant Findings

Contrast-enhanced CT soft tissue of the neck showed evidence of a prevertebral/retropharyngeal fluid collection, extending from the odontoid tip to the inferior C4 vertebral body margin, measuring 5.4 × 1.0 × 3.3 centimeters (cm) in size (yellow lines) without gross airway narrowing. Of note, there was not specific mention of central hypodensity/low attenuation or peripheral enhancement of the abscess rim within the radiology report, which is thought to be particularly helpful in differentiating retropharyngeal abscess from non-infectious retropharyngeal edema.^3^ The presence of rim enhancement provides a sensitivity of 89% for detecting RPA.^4^ There was also evidence of edema within the soft tissues of the lower left neck extending into the axilla and upper chest, as well as multiple mildly enlarged cervical chain lymph nodes bilaterally, which are largely nonspecific findings that may further support the presence of an infectious etiology with soft tissue spread. There was no evidence of cervical vessel involvement, which lowered concern for Lemierre’s Syndrome.

Contrast-enhanced CT chest showed evidence of mild soft tissue edema at the base of the left neck with extension into the superficial soft tissues of the upper chest, also suggesting evidence of a probable retropharyngeal or prevertebral abscess with spread into the soft tissues of the neck base and upper chest. There was no visualized soft tissue fluid collection in the chest or other mediastinal findings.

## Patient Course

The patient’s lab work demonstrated a normal white blood cell count of 7.8 thousand (K)/microliter (uL), normal hemoglobin and hematocrit, and a thrombocytosis of 521K/uL. She had normal electrolytes, lipase, and negative troponin level. Her ECG was within normal limits. After the CT imaging returned with the notable findings, blood cultures and lactic acid were obtained which also returned within normal limits. She remained stable for the duration of her stay in the ED prior to transfer. The patient was started on broad-spectrum intravenous (IV) antibiotics (vancomycin and piperacillin-sulbactam) empirically. She did not require or receive any antipyretics prior to arrival or while under our care. The patient was transferred to another accepting facility for otolaryngologist (ENT) consultation and admission for further monitoring. While there, she received IV antibiotics and steroids on a scheduled basis. She had a flexible laryngoscopy performed by ENT which was unremarkable. After approximately 24 hours, the patient was noted to have symptomatic improvement and was discharged on a two-week course of oral amoxicillin-clavulanate with ENT follow up in two weeks.

A wide differential was considered for our patient presenting with a report of shoulder and neck swelling with odynophagia. Although she had no fever or leukocytosis, infectious pathologies such as retropharyngeal abscess, parapharyngeal abscess, prevertebral abscess, mediastinitis, and Lemierre’s syndrome were considered. Allergic/autoimmune triggers including anaphylaxis and hereditary angioedema/C1 esterase deficiency were also strongly considered with the association of wheezing and abdominal pain. Referred abdominal pain causing shoulder pain, cardiac disease such as acute coronary syndrome, malignancy, and more common complaints such as asthma exacerbation, and GERD were also considered. The progressive odynophagia and focal subjective left-sided neck and chest pain were symptoms that prompted more focus on a neck or upper chest source, especially when considering the continuity of the retropharyngeal space between fascial planes of the neck extending inferiorly to the posterior mediastinum.^5^ These particular symptoms generated concern for a possible space-occupying pathology along these fascial planes. Coupled with the notion that RPA is a consequential diagnosis, we ordered a CT scan before arriving at an alternative diagnosis and management plan. Given a lack of fever and leukocytosis, which is often present in patients with RPA, her clinical presentation was somewhat atypical for RPA and may not have otherwise warranted further investigation via imaging studies.^6^ However, establishing a low threshold for obtaining CT imaging in our patient ultimately revealed the source of her symptoms and allowed us to confirm the diagnosis.

## Discussion

Prompt diagnosis and treatment of RPA in the adult patient can pose a challenge for clinicians, especially in adults and patients presenting with atypical or nonspecific features. Nevertheless, it is imperative to consider RPA as a potential etiology of neck pain, neck swelling, and dysphagia because the consequence of missing this diagnosis may result in significant complications and a higher risk of mortality. Complications of RPA can include airway obstruction, sepsis, acute necrotizing mediastinitis, aspiration pneumonia, epidural abscess, Lemierre’s syndrome, necrotizing fasciitis, and erosions into the carotid artery.^7^ Patients with a concerning presentation should be referred for imaging, preferably via contrast-enhanced CT imaging, to evaluate for RPA before determining a patient’s disposition.

The diagnosis of RPA is alerted by clinical symptoms and confirmed by imaging studies.^7^ In children, lateral neck radiographs are the recommended initial study of choice to reduce radiation exposure and may show evidence of prevertebral space widening [greater than 6 millimeters (mm) at the level of C3 or greater than 14 mm at C6 (in children) or 2 mm at C6 (in adults)] with anterior displacement of pharyngeal structures.^8^ However, there is ongoing debate regarding the sensitivity of lateral neck radiographs and their usefulness in detecting RPA, especially considering the widespread availability of more definitive diagnostic testing.^9^ A CT of the neck with intravenous contrast may be a more definitive study to confirm RPA in stable patients. Studies have shown a sensitivity of 81%, but a specificity of only 45–65% for detecting RPA on contrast-enhanced CT (CECT).^10^ Other studies have reported a false positive rate of 10% and false negative rate of 13% for the detection of purulent fluid within the prevertebral/retropharyngeal space on CECT in pediatric patients.^11^ One study revealed superiority of MRI compared to CT for anatomical discrimination and is thought to be particularly helpful for patients with an equivocal CT, though the time requirement to obtain an MRI may limit its usefulness in practice.^12^ While CT is a useful modality to assist diagnosing cases of RPA, its limited specificity raises concern for false positives, such as in cases of retropharyngeal calcific tendinitis (RCT), which can also manifest as neck pain and retropharyngeal edema on CECT.^13^ Therefore, these results must be considered in conjunction with physical exam findings, laboratory studies, and a thorough patient history. Laboratory studies are often remarkable for leukocytosis greater than 12K/uL in the majority of patients, and blood cultures and preoperative labs should be obtained in all patients with suspected RPA.^14^

Management of RPA is centered on timely recognition and close airway monitoring, initiating broad spectrum antibiotics, and ENT or maxillofacial surgery consult for possible intervention (needle aspiration versus surgical drainage).^14^ In patients requiring emergent airway management, awake fiberoptic intubation can be a beneficial method to prevent further airway obstruction from retropharyngeal edema or possible abscess rupture.^15^ Studies indicate that patients with significant airway obstruction or mass effect are likely to benefit from abscess drainage, typically via a surgical approach performed by an otolaryngologist.^16^ Patients with a milder presentation consisting of smaller abscess (<2.5 cm) without airway obstruction or significant mass effect can be admitted and managed conservatively with a trial of intravenous antibiotics for 24–48 hours with an endpoint goal of symptomatic improvement.^16^ Patients on conservative management without improvement at 24–48 hours should undergo repeat imaging and be reassessed for possible drainage.^16^

Choice of empiric antibiotic should center on providing polymicrobial coverage, including coverage for oral anaerobes. Potential regimens include ampicillin/sulbactam, clindamycin, ceftriaxone or levofloxacin plus metronidazole for patients with known immunocompetency, or vancomycin plus piperacillin/tazobactam for patients with potential or unknown immunocompromise.^16^,^17^ Some studies have shown benefit to initiating corticosteroids for RPA, indicating there may be an association between corticosteroid use and decreased need for surgical drainage and lower hospital costs.^18^ However, the majority of these studies were conducted with pediatric patients; therefore, further studies are needed to assess the efficacy of corticosteroid use in conjunction with standard of care treatments for RPA in adult patients.

In summary, retropharyngeal abscess is a “cannot-miss” diagnosis that must be considered in the differential of adults presenting with symptoms such as fever, neck pain, neck swelling, dysphagia, odynophagia, drooling, trismus, voice changes, and/or respiratory distress. This case report exemplifies the importance of considering imaging for further workup in cases where RPA cannot be excluded from a differential, especially with the presence of atypical features, because timely management is critical towards preventing complications and poor outcomes

## Supplementary Information












