# Supplementary figures and images for: Retropharyngeal Abscess in an Adult Patient Presenting with Neck Fullness and Dysphagia: A Case Report
Source: J Educ Teach Emerg Med. 2025 Jan 31;10(1):V12–6. doi: 10.21980/J8M36G (PMC11801486; doi:10.21980/J8M36G)

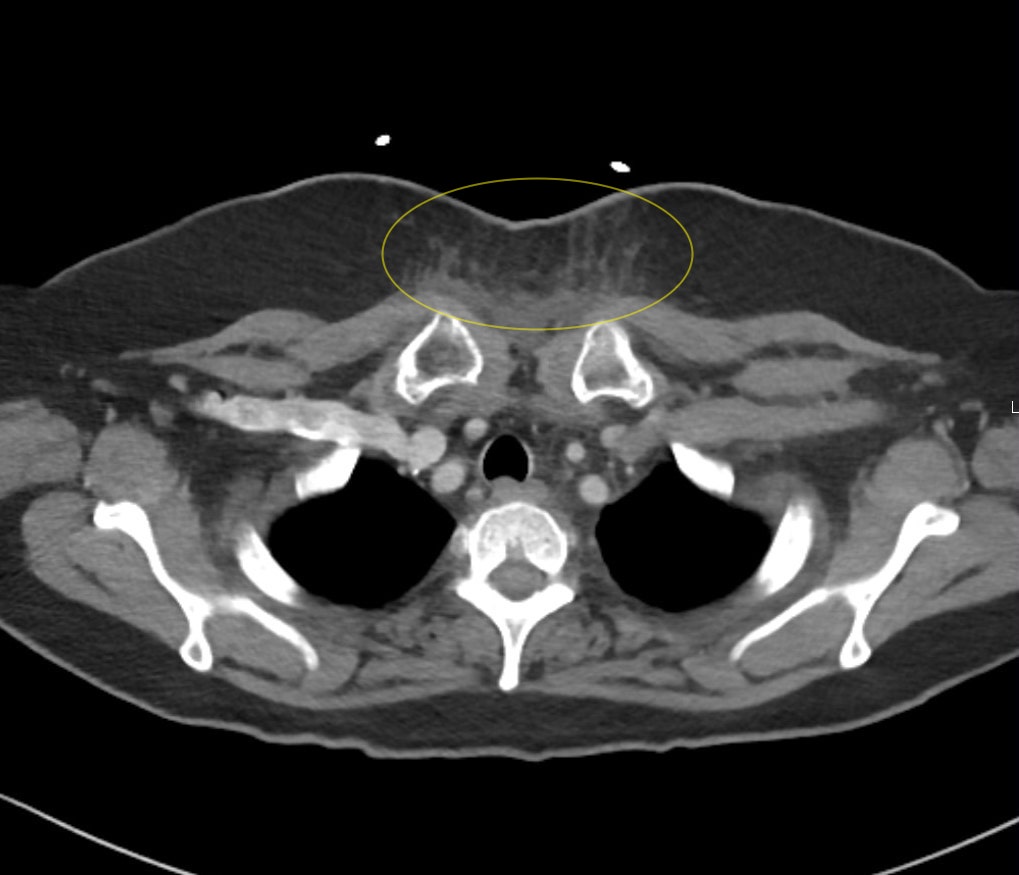

Supplement: Supplementary file 1 [file 10-1-V12-supp1.jpeg]

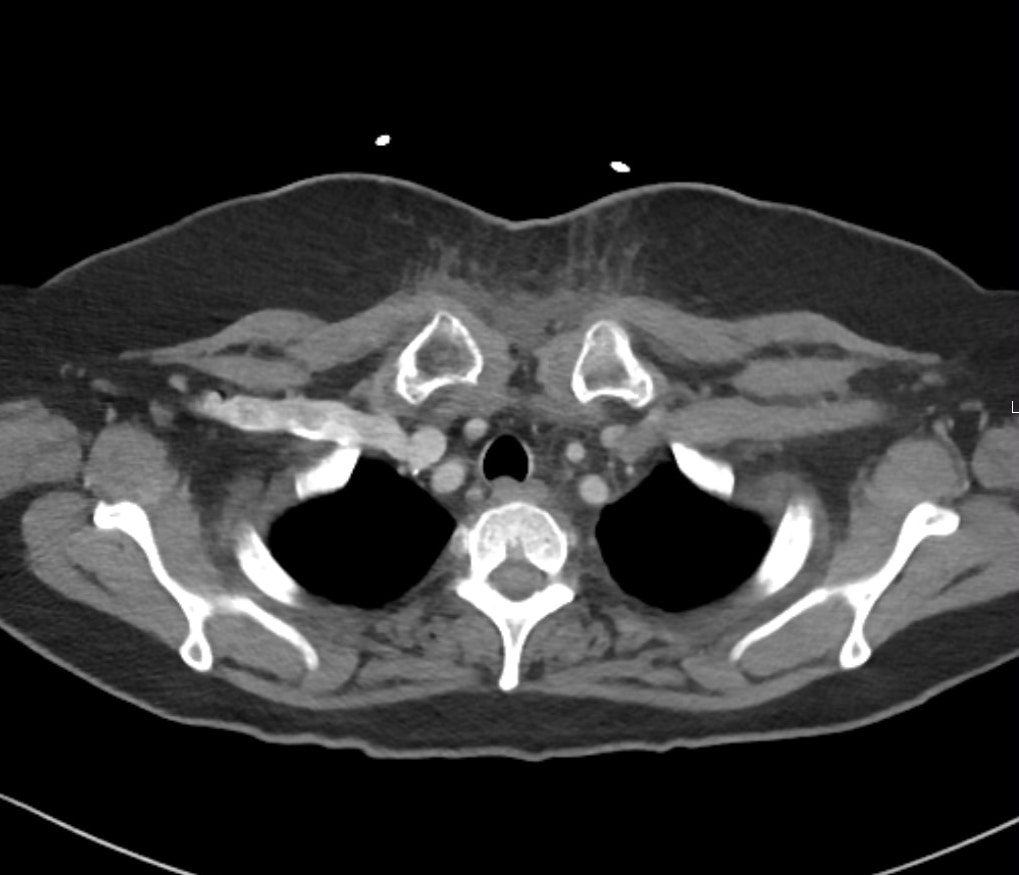

Supplement: Supplementary file 2 [file 10-1-V12-supp2.jpg]

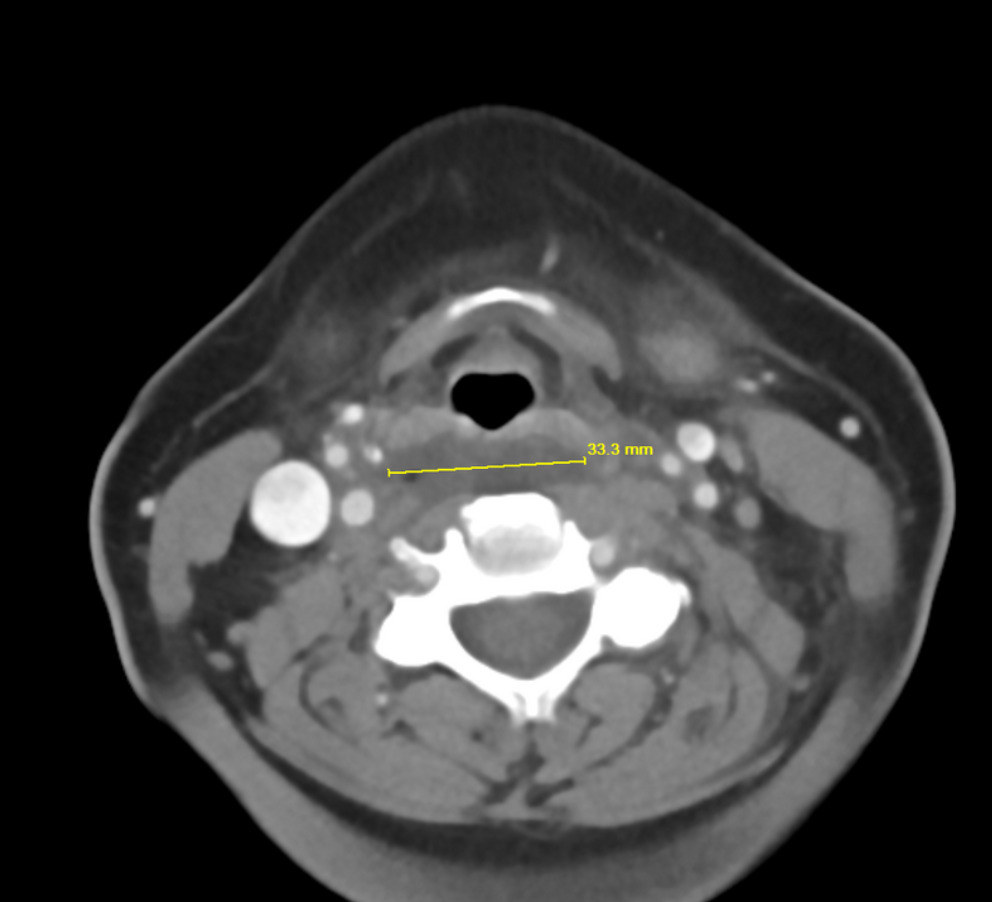

Supplement: Supplementary file 3 [file 10-1-V12-supp3.jpeg]

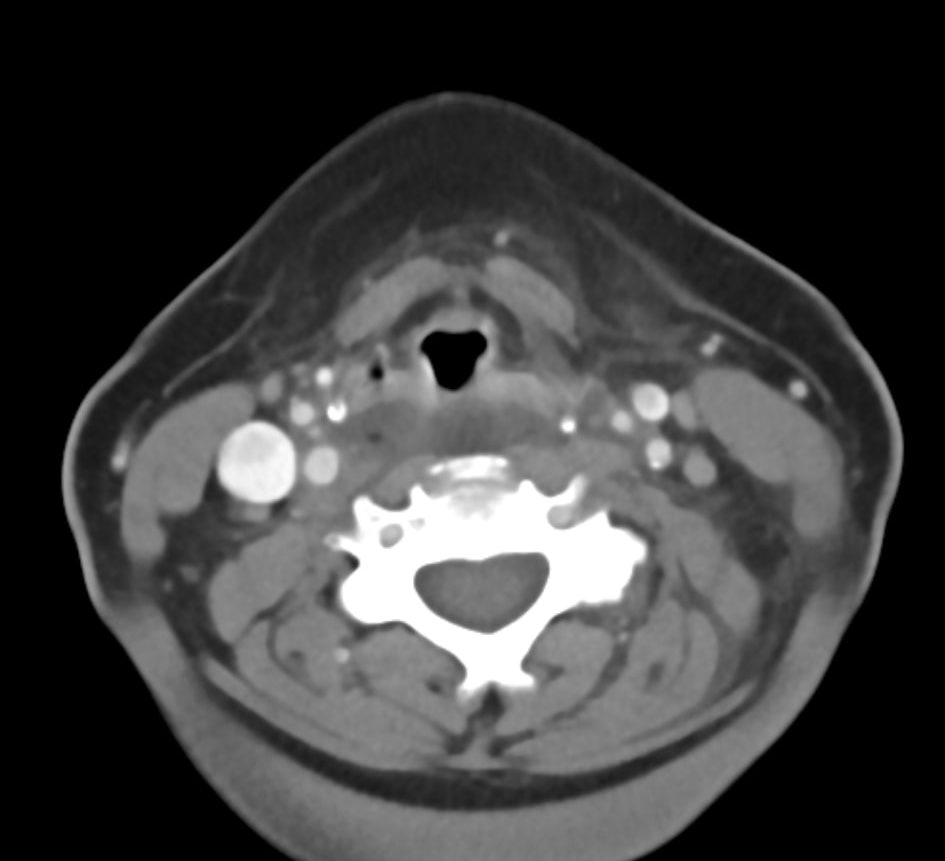

Supplement: Supplementary file 4 [file 10-1-V12-supp4.jpeg]

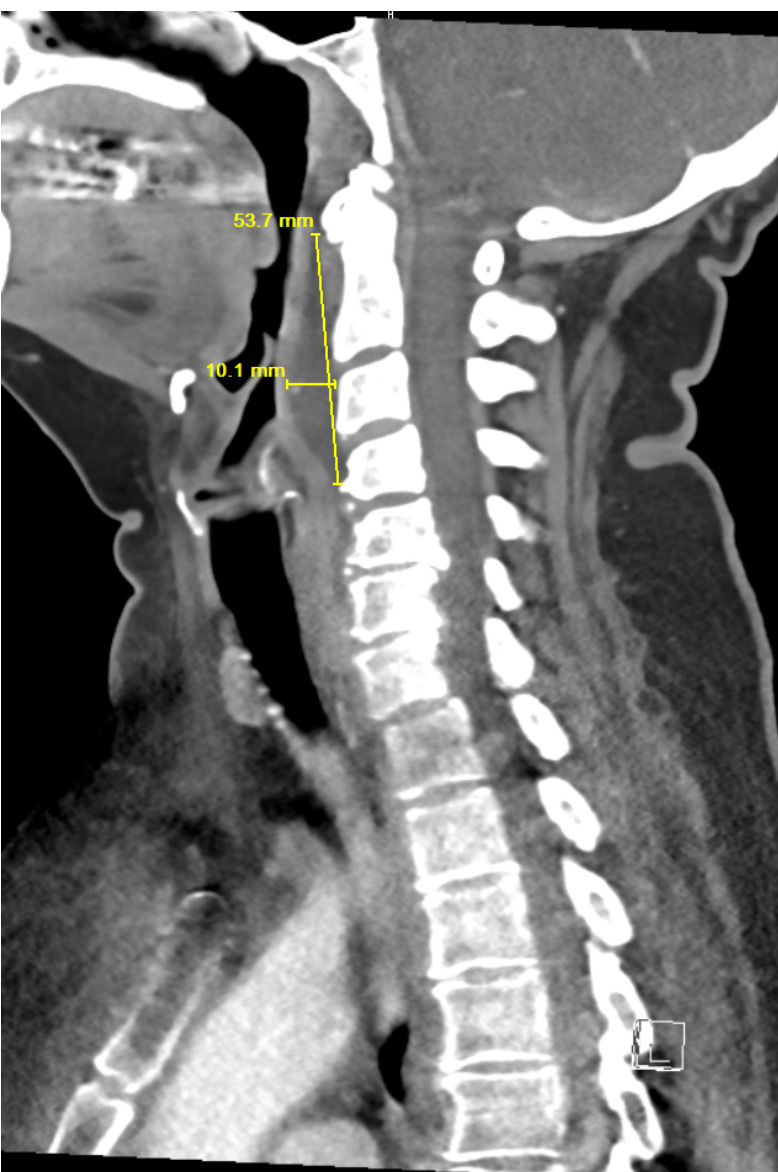

Supplement: Supplementary file 5 [file 10-1-V12-supp5.jpg]

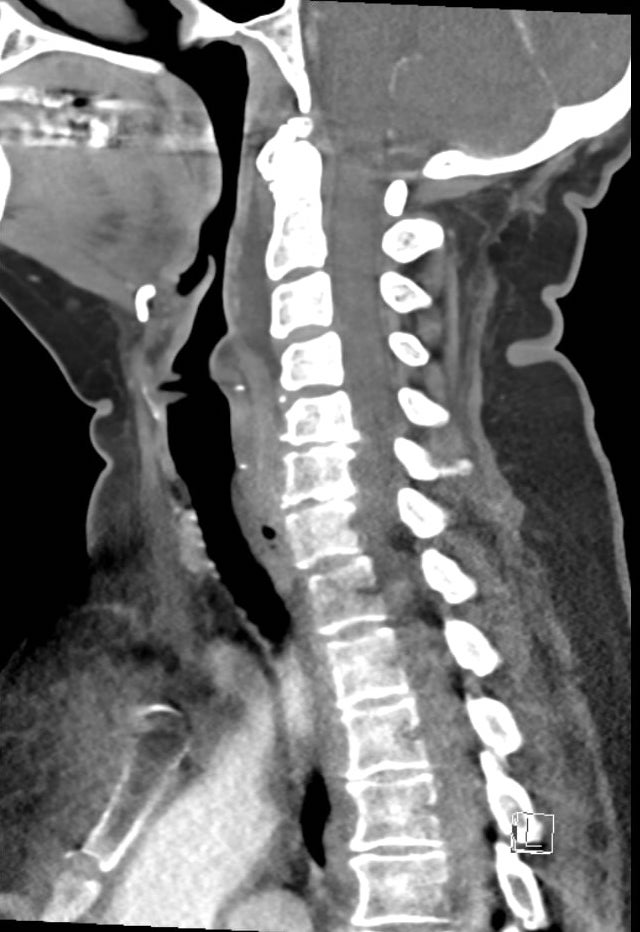

Supplement: Supplementary file 6 [file 10-1-V12-supp6.jpeg]
